# Considerations for Conservative, All-Ceramic Prosthodontic Single-Tooth Replacements in the Anterior Region: A Systematic Review

**DOI:** 10.3390/dj13050219

**Published:** 2025-05-19

**Authors:** Dubravka Knezović Zlatarić, Mirko Soldo

**Affiliations:** 1 Department of Prosthodontics, School of Dental Medicine, University of Zagreb, 10000 Zagreb, Croatia; 2 Community Health Center Osječko-baranjska County, 31000 Osijek, Croatia; ortodoncija1.osijek@dzobz.hr

**Keywords:** resin-bonded fixed dental prosthesis, three-unit fixed partial denture, anterior teeth, survival rate, success rate

## Abstract

**Background/Objectives:** Conservative options for single-tooth replacements in the anterior region include all-ceramic one-retainer resin-bonded fixed dental prostheses (RBFDPs) and three-unit fixed partial dentures (FPDs). **Methods:** This systematic review assessed their clinical outcomes. Following the PRISMA 2020 guidelines, an electronic search of MEDLINE/PubMed was conducted from November 1991 to March 2025 for randomized clinical trials (RCTs), prospective cohort studies (PCSs), and retrospective cohort studies (RCSs). Keywords included dental prosthesis, fixed prosthesis, resin-bonded prosthesis, single-tooth replacement, anterior teeth, all-ceramic, lithium disilicate, monolithic, zirconia, survival rate, and success rate. Failures and complications were analyzed to determine long-term outcomes. **Results:** The search identified 990 articles, and the full-text review of 54 articles was performed, resulting in 23 studies meeting the inclusion criteria. This review revealed that one-retainer RBFDPs and three-unit FPDs in the anterior region demonstrated high survival and success rates. However, debonding was a common complication in RBFDPs, while framework design issues were noted in FPDs. **Conclusions:** These outcomes highlight the reliability of both approaches as conservative, all-ceramic, prosthodontic interventions for anterior single-tooth replacements. The consideration of one-retainer RBFDPs and three-unit FPDs is advisable due to their favorable clinical performance and minimal invasiveness.

## 1. Introduction

The loss of a single tooth in the anterior region has always been one of the most demanding challenges in dentistry, requiring the restoration of both esthetic and functional aspects of the dentition. Various dental conditions may be the reason for missing teeth in this particular region. In the case of congenitally missing teeth, maxillary lateral incisors are the most affected teeth in the anterior region, most often bilaterally [1,2,3]. The same is true for teeth lost due to traumatic dental injuries, where injuries to the maxillary central and lateral incisors are the most common, though the loss is usually unilateral [4,5]. Finally, dental caries and periodontal diseases are some of the main reasons for anterior tooth extractions [6,7]. Aside from esthetic concerns, patients with missing front teeth may suffer from various difficulties like malocclusion, reduced masticatory ability, periodontal damage, alveolar bone loss, inarticulate pronunciation, and psychological problems [8]. In such situations, different treatment options for the replacement of a single missing anterior tooth exist. In addition to treatments intended mostly for growing patients and younger adults such as tooth auto-transplantation and orthodontic space closure, treatments like implant-supported prosthesis (ISP), fixed partial denture (FPD), and resin-bonded fixed dental prosthesis (RBFDP) may be considered in adults [9,10,11,12].

Although ISPs offer significant advantages because they prevent the needless restoration of the sound tooth/teeth adjacent to the edentulous area and therefore provide the opportunity to preserve the integrity of the existing teeth in the situations where implants are surgically contraindicated due to a deficiency in soft and bone tissues, the patient’s serious health issues, or even personal preferences, tooth-supported restorations should be considered [13]. The restorations within the more conservative tooth-supported group differ in terms of the amount of tooth structure removed, with RBFDPs reducing the risk of endodontic complications and therefore corresponding to a minimally invasive treatment alternative and FPD being the most invasive one [14]. Two different types of materials have been used successfully for the fabrication of tooth-supported restorations for decades; however, with patients’ increasing focus on the esthetics of the frontal area, all-ceramic options are increasingly in demand [15].

The rationale of this review was to report clinical procedures together with survival rates of specifically designed all-ceramic one-retainer RBFDPs and three-unit FPDs in the anterior region. None of the previously published literature reviews have combined these two different designs and reported all the details required for searching solutions when it comes to single-tooth replacement in the anterior region. When deciding about the specific design of RBFDPs, the authors were guided by previously reported findings that recommended one-retainer RBFDPs due to their fewer clinical complications [16,17].

The aim of this study was to systematically review all the clinical articles about all-ceramic one-retainer RBFDPs and three-unit FPDs in the anterior region and assess their specific study design, number of restorations, materials and techniques used, observation time, survival, success/failure rates, and modes of failure.

## 2. Materials and Methods

### 2.1. Study Design

Initially, this study was conducted as an exploratory investigation and was not registered in PROSPERO. As the research later evolved into a systematic review, it became apparent that a PROSPERO registration, which typically occurs at the start of this type of study, was no longer feasible.

### 2.2. Systematic Search Strategy

Based on the PICO criteria, a search strategy was developed and executed using an electronic search. The PICO (problem, intervention, control, outcome) question was as follows: “Which design, out of one-retainer all-ceramic RBFDP and three-unit conventional all-ceramic FPD, has a higher survival and success rate for conservative, all-ceramic prosthodontic single-tooth replacement in the anterior region?”. In accordance with the research question, PICO framework was customized and defined as follows:

- P (problem): single-tooth replacement; anterior teeth.
- I (intervention): resin-bonded fixed partial denture; cantilever fixed dental prosthesis.
- C (comparison): three-unit prosthesis; three-unit prosthesis; all-ceramic; lithium disilicate; monolithic; zirconia fixed dental prosthesis.
- O (outcome): survival [MeSH]; success.

**Figure 1:**
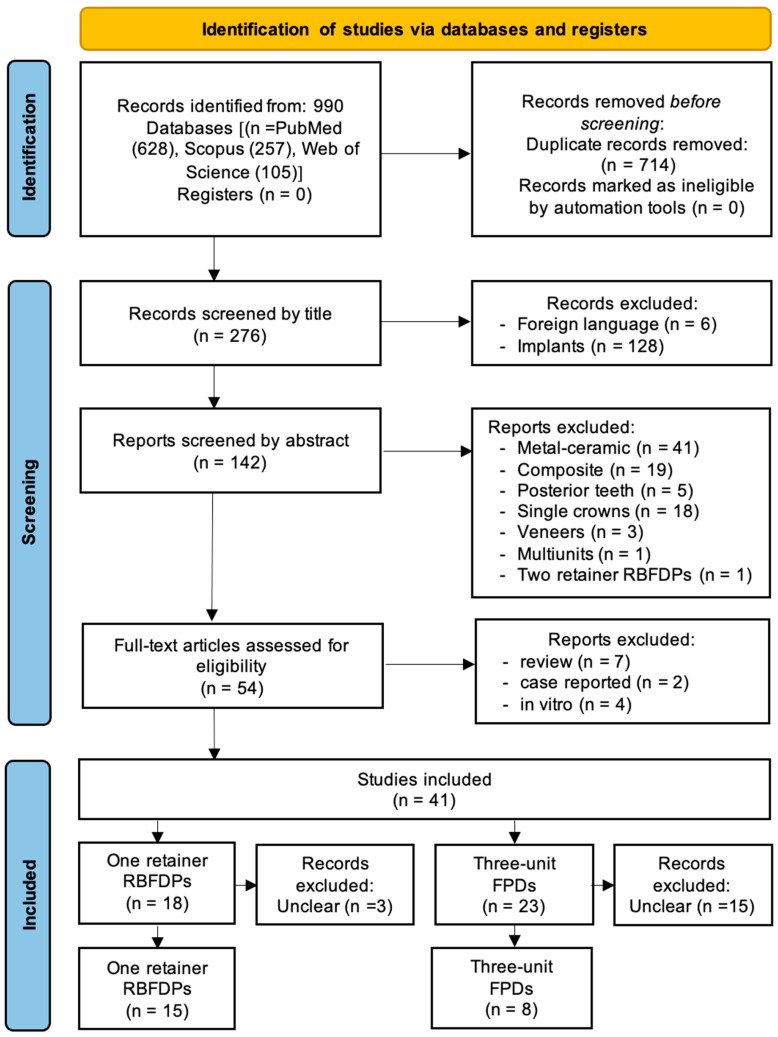
The PRISMA flowchart for the literature search employed for this review.

This review follows the newest updated guideline of the Preferred Reporting Items for Systematic Reviews (PRISMA) 2020 statement [18]. This review was designed according to the updated PRISMA 2020 protocol checklist (available as Supplementary Materials). The literature on RBFDPs and three-unit FPDs was systematically reviewed, and studies were selected following the workflow stages shown in Figure 1.

In the first stage, the databases MEDLINE/PubMed, Scopus, and Web of Science were electronically searched for English language articles in the dental literature published between November 1991 and March 2025. Search was performed using search terms (grouped into PICO categories), and the search strategy was assembled from a combination of qualified Medical Subject Headings (MeSH Terms) as well as nonspecific free-text words in simple or multiple conjunctions as presented in Table 1. The gray literature and manual searches were not conducted.

### 2.3. Study Selection

Potentially relevant articles were selected by reviewing their titles. The inclusion criteria for the studies were as follows:

- Studies in English.
- Treatment concepts with one-retainer all-ceramic RBFDPs and three-unit conventional all-ceramic FPDs in the anterior region.
- Clinical studies only.
- Studies with a mean follow-up of at least 1 year.
- Studies reporting on details and outcomes of selected treatments.

Based on the defined inclusion criteria, titles were retrieved with a systematic search and independently screened by two reviewers (authors). Inter-rater reliability was assessed using Cohen’s kappa statistic, yielding a value of 0.82 for study selection and 0.78 for data extraction, indicating substantial agreement. To avoid any bias during the selection, disagreements between the two authors during study selection were resolved through structured discussion until a consensus was reached, ensuring an unbiased and consistent selection process.

### 2.4. Data Extraction

The following information was obtained from the included publications: the description of the specific study design, the number of restorations, materials and techniques used, observation time, survival, success/failure rates, and the modes of failure.

### 2.5. Outcome Measures

The primary outcome of this review was the survival and success data of both observed groups. Survival was defined as RBFDPs or three-unit FPDs remaining in situ for the observation period with or without modifications. The evaluation of biological considerations included soft tissue inflammations, tooth mobility (including rotations), caries, and tooth pain. Finally, technical considerations included debonding events (successfully rebounded during the observation period), chipping of the veneering ceramic, framework fracture, or total loss of the restoration.

### 2.6. Effect Measures for Synthesis

To compare the outcomes, the primary effect measures used were as follows:

- Survival Rates: the percentage of restorations that remained in situ without major complications during the observation period in both the RBFDP and three-unit FPD groups.
- Success Rates: the percentage of restorations that remained functional, with minimal complications (such as debonding, chipping, or fractures) during the observation period in both groups.

These effect measures allowed for a direct comparison of the survival and success rates between the two types of restorations over time.

### 2.7. Risk-of-Bias Assessment

Both authors independently performed a risk-of-bias analysis of all included studies according to the revised Cochrane risk-of-bias tool for randomized trials (RoB2) (5 domains) and the Methodological Index for Non-Randomized Studies (MINORS) (8 domains) [19,20].

The RoB 2 tool was applied to randomized controlled trials to evaluate bias across five domains (randomization process, deviations from intended interventions, missing outcome data, measurement of the outcome, and selection of the reported result), while the MINORS tool was used for non-randomized studies, assessing 8 methodological criteria (including a clearly stated aim, prospective data collection, appropriate endpoints, unbiased assessment, and follow-up), with each item scored according to the standard guidelines for each tool (low, some concerns, high).

In general, all included clinical studies showed some concerns, being not completely bias-free. Studies were rated with a low-to-moderate risk of bias.

### 2.8. Data Synthesis

Substantial heterogeneity was present across the studies, reflected in differences in study designs (randomized clinical trials, and retrospective and prospective studies), outcome measures (failure rates, retention rates, and satisfactory rates), types of restorations assessed, inclusion of both anterior and posterior restorations, variability in observation periods, and the need for recalculation and extraction of data due to inconsistent reporting. Since no specific statistical synthesis (e.g., meta-analysis) was conducted, the outcomes of interest were compared descriptively. The primary comparison was based on the survival and success rates between the one-retainer all-ceramic RBFDPs and the three-unit conventional all-ceramic FPDs. These outcomes were reported for each study, and a qualitative synthesis was performed to compare the survival and success rates between the two types of prostheses. They addressed the observed heterogeneity in study methodologies and definitions of outcomes, and a narrative exploration of possible sources of inconsistency was undertaken. Sensitivity analyses to assess the robustness of synthesized results were not applicable.

## 3. Results

### 3.1. Included Studies

The initial electronic search of the research database yielded 990 articles published between November 1991 and March 2025. Following the initial elimination of duplicates, 276 titles were identified and screened; subsequently, 134 titles were excluded. The reasons for exclusion were as follows:

- Studies written in foreign languages (n = 6).
- Studies on implant-supported all-ceramic restorations (n = 128).

For the 142 relevant titles, the abstracts were screened, and 88 titles were included for further evaluation. The exclusion criteria were as follows:

- Use of composite or metal–ceramic restorations (n = 60).
- Studies conducted on posterior teeth (n = 5).
- Studies employing different treatment concepts (n = 23) (Table 2).

Eventually, 54 full articles were selected for detailed review. Thirteen articles were further excluded due to their unacceptable study design. Out of the 41 full-text articles assessed, 18 were initially considered eligible but were ultimately excluded. While these studies might have initially appeared to meet the inclusion criteria, they were excluded due to issues such as insufficient details on restoration design, span length, or anatomical location, making it impossible to differentiate between anterior and posterior placements or to isolate specific restoration types such as three-unit fixed partial dentures. In many cases, the data were presented in aggregated formats without clear categorization, hindering meaningful interpretation and targeted data extraction.

Overall, after screening over 900 potential articles, 23 clinical studies met the inclusion criteria and were subsequently categorized according to different treatment concepts investigated in this systematic review, i.e., 15 in the RBFDP group and 8 in the three-unit FPD group, shown in the flowchart in Figure 1.

### 3.2. Descriptive Analysis

From the 623 identified articles, 15 met the inclusion criteria for the RBFDP group and 8 for the three-unit FPD group. The following information was gathered from each article’s review—the number of restorations, material, technique, observation time, survival and failure/success rates, and modes of failure.

### 3.3. RBFDPs

The screening of the selected articles according to the inclusion and exclusion criteria resulted in 15 original studies in this group: 4 RCTs, 1 clinical controlled trial (CCT), 6 PCSs, and 4 RCSs (presented in Table 3). The number of restorations ranged from 10 to 108.

Different materials and techniques were employed in these studies. In three studies, RBFDPs were fabricated using glass-infiltrated ceramic In-Ceram alumina or In-Ceram zirconia with a copy-milling technique and veneered with Vitadur-Alpha (Vita; Bad Säckingen, Germany). In one study, a slip casting technique was applied for In-Ceram alumina, and the restorations were veneered using VM7; another study used In-Ceram alumina, but the specific technique was not detailed (Vita; Bad Säckingen, Germany). Two studies utilized IPS e.max ZirCAD and IPS e.max Ceram in a milled/veneered technique, while another two studies employed the same material with GC Initial (Ivoclar; Schaan; Liechtenstein; GC; Tokyo; Japan). One study compared the two materials in a milled/veneered technique: Zenotec (Wieland Dental + Technik GmbH & Co., KG; Pforzheim, Germany)/Zirox (Wieland Dental + Technik GmbH & Co., KG; Pforzheim, Germany) and Lava Plus (3M Espe)/IPS e.max Ceram (Ivoclar; Schaan; Liechtenstein). Another study used four different zirconia and three different veneering materials in a milled/veneered technique, and three studies did not specify the zirconia material used. One study described the use of IPS e.max Press or IPS Empress with IPS e.max Ceram in a pressed/veneered technique.

The mean observation time ranged from 34 to 188 months, with survival rates extending between 81.8% and 100%. Reported failures in the studies included 27 debondings, 2 chippings, 2 tooth rotations, 1 tooth movement, and 9 fractures, and 1 RBFDP was removed at the patient’s request: an implant-supported crown after a minor chip on the mesial edge of the RBFDP pontic.

### 3.4. Three-Unit FPDs

Of the eight original studies in this group, seven were PCSs and one was an RCS (presented in Table 4). The number of restorations ranged from 3 to 16. In two studies, FPDs were fabricated from In-Ceram alumina and Vitadur Alpha using a slip casting/veneered technique. Three studies used IPS e.max or Empress 2 in a pressed monolithic technique and two utilized Lava/Lava Ceram or KaVo Everest ZS/IPS e.max Ceram in a milled/veneered technique. One study used IPS e.max CAD LT.

The duration of observation in the studies varied, with a range of 16 to 120 months. Survival rates were reported to be between 81.3% and 100%. The documented failures included cases of localized mild gingivitis, three instances of ceramic chipping, six occurrences of framework fractures, a single total loss of a three-unit FPD attributable to secondary caries, and two losses involving persistent pain despite endodontic treatments.

### 3.5. Synthesis of Results

Survival rates were influenced by the length of observation periods, with shorter durations generally yielding higher survival percentages. The variability in materials and techniques across studies further contributed to the observed heterogeneity, emphasizing the need for standardized reporting in future research.

### 3.6. Reporting Bias

**Figure 2:**
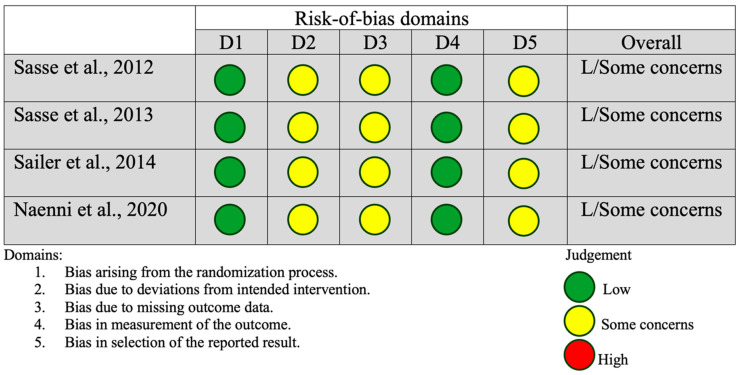
The risk-of-bias assessment summary according to the revised Cochrane risk-of-bias tool for randomized trials (Rob2) [19,21,22,24,30].

**Figure 3:**
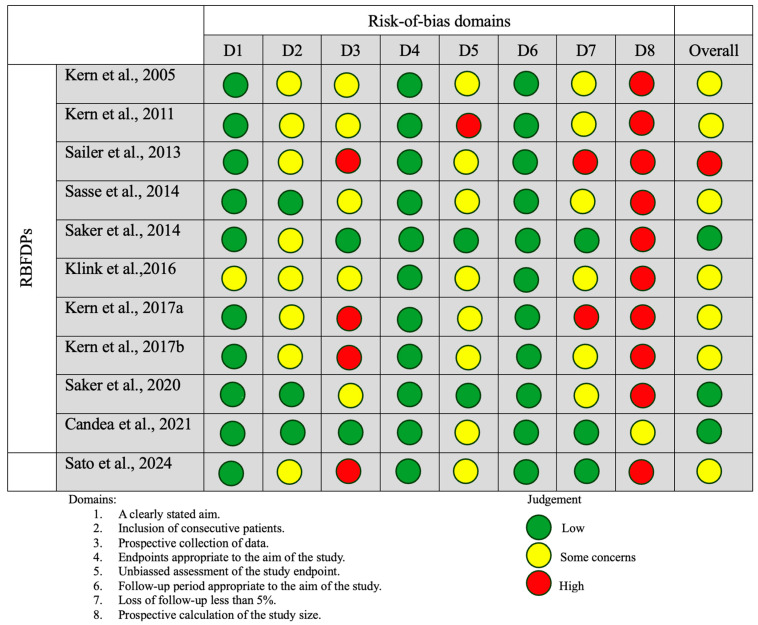
The risk-of-bias assessment summary according to the methodological index for non-randomized studies in the RBFDP group (MINORS) [16,17,20,23,25,26,27,28,29,31,32,33].

**Figure 4:**
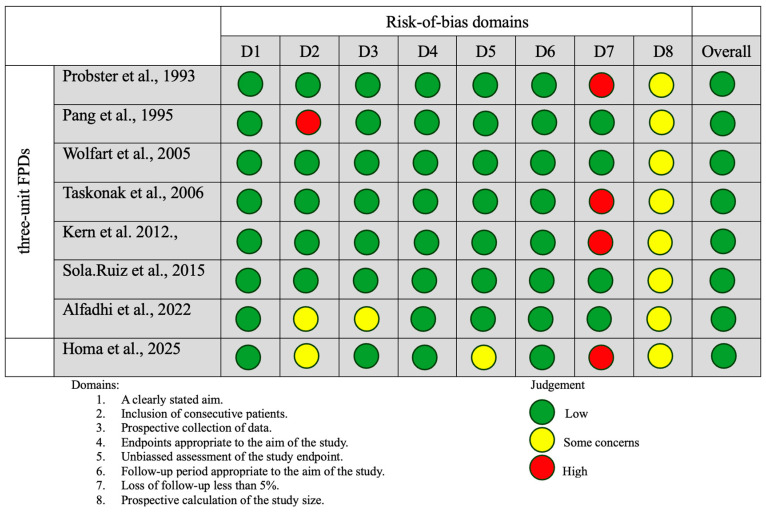
The risk-of-bias assessment summary according to the methodological index for non-randomized studies in three-unit FPD group (MINORS) [20,34,35,36,37,38,39,40,41].

An assessment of reporting bias due to missing results for both the RBFDP and three-unit FPD groups indicated a low-to-moderate risk. The risk-of-bias assessment summary according to the revised Cochrane risk-of-bias tool for randomized trials (RoB 2) indicated that while the studies may be generally free from significant bias in certain areas (such as patient selection and follow-up), there was some uncertainty regarding other potential sources of bias, particularly in the reporting and assessment of outcomes (Figure 2). Non-randomized studies in the RBFDP group generally demonstrated a low risk of bias in key domains such as patient selection and follow-up. However, there remained some concern regarding potential bias in outcome assessment and reporting, where several studies lacked sufficient detail to allow for a clear judgment. This indicated that while the overall methodological quality is acceptable, caution was warranted when interpreting the reported outcomes (Figure 3). Non-randomized studies in the FPD group generally had a low to moderate risk of bias, with strengths in clear aims, prospective data collection, and unbiased assessments. Common weaknesses included unclear or small sample size calculations and, in a few cases, non-consecutive patient recruitment or minor loss to follow-up (Figure 4). Despite that some studies had incomplete reporting of outcomes this did not appear to substantially impact the overall findings (Figure 2, Figure 3 and Figure 4).

### 3.7. Certainty of Evidence

The certainty of the evidence for each outcome was assessed based on the risk of bias and other study limitations. For the RBFDP group, the certainty of evidence was considered moderate for most outcomes, primarily due to factors such as a higher risk of bias in several studies, small sample sizes, and imprecision in some reported outcomes. Similarly, for the three-unit FPD group, the certainty was moderate, with limitations related to inconsistent results across studies, incomplete reporting of key outcomes, and a higher risk of bias in some included studies.

## 4. Discussion

In general, numerous shortcomings were identified across all studies within the systematic review framework. A comprehensive analysis of the literature revealed limited evidence stemming from the study designs employed when evaluating conservative treatment modalities for the restoration of a single anterior tooth utilizing all-ceramic materials. RCTs present a viable approach for directly comparing the efficacy of one-retainer RBFDPs and FPDs. However, the absence of such comparative studies hampers the ability to draw definitive conclusions. Criticism directed towards the existing literature primarily arises from the absence of randomized clinical trials directly comparing these two designs, necessitating an indirect comparison of studies focusing on each treatment modality individually. Consequently, there emerges a lack of consistency in the evaluation of survival and success/failure rates, primarily due to differing criteria employed for assessing outcomes between RBFDP and FPD treatments, where the former predominantly considers debonding events while the latter focuses more on framework fractures. Considering that the systematic reviews of RCTs offer the highest level of evidence containing the least amount of bias (which has been taken into consideration in this review), very few RCTs were found, only four in the RBFDP group and none in the three-unit FPD group [21,22,24,30]. The lack of high-quality randomized studies for this particular review could be attributed to a common problem in biomedical research: these types of clinical trials are usually very expensive to carry out, time-consuming, and require resources that are rarely accessible to the majority of independent investigators [42,43].

For this systematic review, MEDLINE (PubMed), Scopus, and Web of Science were selected as the primary databases, given their widespread use and comprehensive coverage of biomedical literature. However, despite this, the availability of RCT articles on the selected research topic was relatively limited. Due to the limited availability of RCTs, this systematic review also included high-quality, non-randomized studies such as CCTs, PCSs, and RCSs. Among these, PCSs appeared to be the most frequent across both groups under observation. Notably, in the group of three-unit FPDs, there was just a single RCS reported [16,17,23,25,26,27,28,29,30,31,32,33,34,35,36,37,38,39,40,41]. Reflecting the findings of our own research, the paper by Tezulas et al. on a topic closely related to this one also highlighted the scarcity of RCTs, with their review of 29 original studies on various designs of RBFDPs in the anterior region including a single RCT with a majority of CCTs, PCSs, RCSs, and different types of clinical reports [44].

The next issue we came across in this research was that a large number of studies included in the systematic review utilized different designs of fixed restorations together, such as one- and two-retainer RBFDPs, anterior and posterior RBFDPs and FPDs, single crowns, and short and long edentulous spans, often employing various materials. This made the interpretation of their statistics complicated, since the factor of interest in this review was the conservative replacement of a single missing tooth specifically in the anterior region; therefore, in some papers considered in this review, specially within the three-unit FPD group, data, if possible, had to be extracted upon calculation, together with final rates. The inability to distinguish between anterior and posterior three-unit FPDs further clarifies the incidence of 15 dropouts in the group during the final stage of elimination. Most studies exclusively examined only one-retainer RBFDPs, tracking the survival rate by assessing technical, biological, and esthetic outcomes or exploring the impact of different bonding systems on these outcomes [21,22,23,24,25,27,28,29,30]. In the rest of the studies from this group, one-retainer RBFDPs comprised a portion of the overall sample and were subjected to comparisons with RBFDPs of different designs (two-retainer RBFDPs) or material (cobalt–chromium, composite) [16,17,26,31,33]. Therefore, for the analysis presented in this systematic review, the necessary data were extracted and subjected to manual calculation. From one specific paper, it was impossible to extract information about the number of anterior one-retainer all-ceramic RBFDPs; however, as the survival rate in the group of both anterior and posterior one-retainer designs reported in the study was 100%, it was deemed relevant and included in the analysis [32].

In the FDP group, a limited number of studies were selected, with no investigations focusing exclusively on the survival of three-unit FPDs being identified. The sample size for this specific group was significantly smaller in comparison to the one-retainer RBFDP group, as all studies conducted comparisons involving the survival of single crowns and three-, four-, five-, or even six-unit restorations, most often in both the anterior and posterior regions [34,35,36,37,38,39,40,41]. When data about the survival of anterior three-unit FPDs were partially unclear in the follow-up time, the survival rate was computed up to the point at which it was explicitly stated in the paper (Table 3).

The materials and techniques employed in both observed groups within this study exhibited notable similarities. Several distinct types of materials characterized by varying chemical compositions (zirconia-, alumina- or silica-based glass-infiltrated or lithium disilicate ceramics), combined with diverse esthetic and mechanical properties commonly encountered in everyday prosthodontic practice, were utilized [45,46,47]. In both observed groups, the materials and techniques used were influenced by the time-based context of publication (Table 2 and Table 3). This involved the application of materials and techniques extending from older methodologies, such as the slip casting of In-Ceram alumina, to more recent advancements integrating the CAD/CAM technology [34,35,36,37,38,39,40,41]. In the context of RBFDPs, the employment of a bilayered technique was uniformly adopted. In contrast, the fabrication of FPDs predominantly relied on the bilayered technique, with the inclusion of a pressed or CAD/CAM monolithic technique described in five of the studies [34,35,36,37,38,39,40,41]. The bilayered method is advocated for anterior restorations due to its capacity to deliver superior esthetic results, particularly in the areas of significant esthetic concern, such as with the conventional replacement of a single anterior teeth—a focus of this systematic review [48]. Moreover, a novel monolithic technique for fabricating prosthodontic restorations has been recently introduced in the field of digital dentistry [49]. However, longitudinal studies that compare the survival rates of these CAD/CAM monolithic restorations with reduced abutment tooth reduction to those of conventional methods are yet to be published and considered for inclusion in systematic reviews. This could likely be attributed to the relatively recent introduction of this minimally invasive technique, which demands a considerable duration to be effectively scientifically assessed and implemented. In this systematic review, only one study (the most recent one) was included that investigated CAD/CAM-manufactured three-unit FDPs after an average observation period of 120 months, which indicates that such studies have been published only recently [41].

The major challenge encountered in this review was the heterogeneity and inconsistency among the primary studies, particularly in the definition, calculation, and interpretation of survival, success, and failure rates. This variability in study methodologies, along with notable attrition in some trials, made direct comparisons difficult and precluded the possibility of conducting a formal meta-analysis. The lack of standardized metrics and diverse participant cohorts further hindered a quantitative synthesis. As a result, the review relied on descriptive analysis, but this limitation underscores the importance of consistent definitions and methodology in future studies. Additionally, potential biases, such as publication bias and data extraction errors, as well as limitations in the comprehensiveness of the literature search, should be acknowledged as further constraints of the current review.

In this study, the survival rates for RBFDPs and FPDs were found to be 81.8–100% [16,17,18,19,20,21,22,23,24,25,26,27,28,29,30,31,32,33] and 81.3–100% [34,35,36,37,38,39,40,41], respectively (Table 3 and Table 4). The data indicated that the outcomes were largely similar between the two groups. However, the duration of observation, extending up to 18 years for the RBFDP group in one study, had a significant impact on these rates [29]. Noteworthily, in instances where the observation period was longer, the survival rates for the RBFDP group dipped below 90%, with specific studies documenting rates of 81.8% and 85% [29,31]. It should be highlighted that, in the majority of studies involving the RBFDP group, the average observational duration was between 4 and 6 years, which correlated with a survival rate exceeding 92% [16,21,22,23,24,25,32]. This outcome is in full agreement with the five-year survival rates for anterior metal–ceramic RBFDPs with the same design, which were reported by Gotfredsen et al. to be 91%, and lower than the 100% reported by Botelho et al. [50,51]. The findings suggest that both framework designs of RBFDPs are highly effective in addressing the issue of a single anterior tooth loss, but the esthetic aspect cannot be overlooked, since the level of patient satisfaction regarding the appearance of these restorations is notably reduced when a metal framework is involved [52]. Studies with shorter observational periods reported higher survival rates [26,27].

In the subset of studies concerning FPDs, which was comparatively smaller, a survival rate of 100% was noted across four studies [34,35,36,38]. In the remaining studies, the recorded survival rates were 90%, 87.6% and 81.3%, respectively [39,40,41]. Noteworthily, studies on FPDs had shorter observational periods, generally not exceeding five years (mostly up to two years), with the exception of two studies where the 90% survival rate corresponded to a seven-year observation period and the 81.3% survival rate corresponded to a ten-year observation period, respectively (Table 4) [39,41]. In this group, it was not possible to compare survival rates with those of studies of a similar design but different materials, like those of anterior metal–ceramic FPDs, as seen in the RBFDP group. This is likely due to the absence of studies on such comparisons, possibly because anterior metal–ceramic bridges lack esthetic indications in the area under consideration.

In evaluating the data regarding success and failure rates, significant challenges arose due to the heterogeneity in reporting across different studies, which mostly related to the RBFDP group (Table 3). Sasse et al. have documented failure-free rates of 93.3% over 3 years and 94.5% over 6 years, alongside a 6-year success rate of 97.4% for zirconia milled/veneered RBFDPs [22,25]. In contrast, Sailer et al. have observed a 5-year chipping rate of 5% for lithium disilicate pressed/veneered RBFDPs, while Saker et al. have calculated an estimated annual failure rate of 0.05% for glass-infiltrated RBFDPs within 3 years [23,26]. Klink et al. determined a success rate of 82.4% at 3 years, while Kern et al. found a success rate of 92% at the 10-year observation for zirconia milled/veneered RBFDPs [27,28]. Additionally, Saker et al. in a separate study documented the clinical retention rate of 70% for glass-infiltrated RBFDPs over a 10-year period [31]. At the five-year evaluation, the success rates for zirconia and glass-reinforced ceramic RBFDPs presented in this systematic review coincide with the outcomes presented by Alraheam et al., who report the estimated five-year success rates for these two materials as 92% and 94.3%, respectively [53]. Nonetheless, it is advisable to approach these results with caution, given that in the referenced study, the differentiation between RBFDPs in the anterior and posterior locations and the framework design (the number of retainers) was not explicitly made, a factor that could potentially influence the overall results [53].

Within the group of FPDs, only two studies reported additional outcomes besides the survival rate. The research conducted by Taskonak et al. reported outcomes of pressed lithium disilicate restorations using satisfaction rates, revealing that, after a two-year evaluation, 50% of the FPDs made of milled lithium disilicate were classified as unsatisfactory (Table 4) [37]. Homa et al. reported the success rate for CAD/CAM milled/veneered restorations at a ten-year follow-up due to one chipping (Table 3) [41].

This systematic review’s analysis of failure causes revealed more consistency within each examined group. Technical complications emerged as the predominant failure mode for both groups. Specifically, the debonding of the framework leading to a loss of retention was primarily noted in the RBFDP group, whereas the fractures of the adhesive frameworks were the main technical complication in the FPD group, with frequencies of 66% and 37.5%, respectively. Notably, debonding was most associated with RBFDPs fabricated from zirconia, with two incidences reported for glass-infiltrated ceramic frameworks [21,22,24,25,26,27,28,30,31,33].

This variability in clinical outcomes may be attributed to the mechanical properties of the materials used as the flexural strength of zirconia significantly surpasses that of both natural teeth and all other examined ceramic types [54,55]. However, Kern et al. reported that all debonded RBFDPs were rebonded successfully with no further complications [28]. Among other technical complications, the occurrences of fractures and chipping in RBFDPs were documented with lower incidences, at 33.3% and 13.3%, respectively [16,17,23,26,27,29,31].

In the FPD group, framework fractures were reported in three studies, with a total of six materials—two zirconia and four lithium disilicate materials [37,40,41]. The comparatively higher incidence of fractures in lithium disilicate may be attributed to its lower fracture resistance under normal masticatory forces as opposed to zirconia [53].

Almost all biological complications identified in this systematic review for the both groups were minor. In the RBFDP group, Sasse et al. treated secondary caries with composite fillings and tooth rotations with thermoformed splints [21,22,25]. Klink et al. reported one tooth movement, and it was retreated by an orthodontist; therefore, the complications did not influence the final result [27]. These results coincide with other systematic review reports where dental caries and periodontal problems were the main biological complications with very low occurrence [53]. In the FPD group, one localized mild gingivitis case (position not clear) was reported, one FPD was removed because of the secondary caries, and two FDPs were removed because of the persistent pain in previously endodontically treated teeth [35,39,41]. This type of complication, more prominent in the FDP group, could be attributed to the fact that this type of restoration is typically performed for teeth that are more severely damaged (sometimes endodontically treated) and may require more extensive preparation; furthermore, resin-bonded fixed dentures are unlikely to be placed on such compromised teeth.

Both one-retainer RBFDPs and three-unit FPDs demonstrated high survival and success rates, although each design exhibited distinct technical complications. These findings highlight the importance of patient-specific considerations in clinical decision making when selecting materials and designs for anterior single-tooth replacements. One-retainer RBFDPs may be more suitable for patients seeking minimal tooth preparation and less invasive treatment, making them a good option for preserving tooth structure in cases with minimal tooth reduction requirements. In contrast, three-unit FPDs may be more appropriate for cases with specific biomechanical needs, such as when additional support and stability are required, especially in patients with more substantial occlusal forces. Future studies should aim to directly compare these designs in randomized controlled trials to validate these observations and guide clinical protocols. Additionally, the long-term outcomes of emerging techniques, such as CAD/CAM monolithic restorations, remain underexplored and warrant further clinical investigation to assess their viability and effectiveness in anterior restorations.

In accordance with the GRADE methodology, the certainty of the evidence in this systematic review was assessed across various domains, including risk of bias, inconsistency, imprecision, and indirectness. The overall quality of the evidence for both one-retainer RBFDPs and three-unit FPDs was rated as moderate. While both types of restorations demonstrated high survival and success rates, the certainty of the evidence was compromised by several factors. These included the limited number of randomized controlled trials (RCTs), the heterogeneity of the included studies, and the variability in study designs and materials. Additionally, differences in outcome measures and patient cohorts contributed to some uncertainty. Despite these limitations, the evidence provided useful insights into the comparative performance of the two restoration types. Future studies should aim to address these gaps by incorporating more robust RCTs with standardized outcomes to enhance the certainty of the evidence and inform clinical decision-making more effectively.

## 5. Conclusions

Although both one-retainer RBFDPs and three-unit FPDs are associated with high survival and success rates, technical complications, such as debonding in RBFDPs and framework fractures in FPDs, remain relatively common. Biological complications were generally minor. However, due to the moderate quality of the available evidence, primarily due to the limited number of randomized controlled trials and variability in study designs, the certainty of the findings is somewhat limited. To better assess the long-term outcomes of one-retainer RBFDPs and three-unit FPDs in the anterior region, further well-designed, randomized controlled trials with larger sample sizes, standardized outcome measures, and extended follow-up periods are recommended. These studies would enhance the certainty of the evidence and help guide clinical decision-making more effectively.

## Figures and Tables

**Table 1:** Search strategies for included databases.

Search Strategy	
MEDLINE via PubMed (advanced research)	Title/Abstract single tooth replacement [Title/Abstract] OR anterior teeth cantilever fixed dental prosthesis [Title/Abstract] OR resin bonded fixed partial denture [Title/Abstract] OR three-unit prosthesis [Title/Abstract] OR 3-unit prosthesis [Title/Abstract] OR all-ceramic [Title/Abstract] OR lithium disilicate [Title/Abstract] OR monolithic [Title/Abstract] OR zirconia fixed dental prosthesis [Title/Abstract]) AND (survival [Title/Abstract] [MeSH] OR success [Title/Abstract])
Scopus (advanced search)	Title-Abstract-Keywords single AND tooth AND Replacement OR anterior AND teeth AND cantilever AND fixed AND dental AND prosthesis OR resin AND bonded AND fixed AND partial AND denture OR three-unit AND prosthesis OR 3-unit AND prosthesis OR all-ceramic OR lithium AND disilicate OR monolithic OR zirconia AND fixed AND dental AND prosthesis AND survival OR success
Web of Science (advanced research)	Keywords (AK = (single tooth replacement OR anterior teeth cantilever fixed dental prosthesis OR resin bonded fixed partial denture OR three-unit prosthesis OR 3-unit prosthesis OR all-ceramic OR lithium disilicate OR monolithic OR zirconia fixed dental prosthesis)) AND AK = (success OR survival)

**Table 2:** The eligibility criteria of the systematic review.

Inclusion Criteria	Exclusion Criteria
Human trials	Animal trials, in vitro studies, preclinical trials
Studies in English	Studies in foreign languages
Study design: clinical studies (RCTs, PCS, RCSs, CCTs)	Study design: case reports, case series, systematic reviews
Studies with a mean follow-up of at least 1 year	Studies with a mean follow-up of less than 1 year
Studies reporting on details and outcomes of selected treatments	Studies employing different treatment concepts
Treatment concepts with one-retainer all-ceramic RBFDPs and three-unit conventional all-ceramic FPDs in the anterior region	Studies on implant-supported all-ceramic restorations and composite or metal–ceramic restorations Studies on single crowns, veneers, splinted, or two-wing RBFDPs Studies conducted on premolars or molars
	Studies with no or insufficient information on survival and success rates/data extraction unavailable

**Table 3:** The details of the articles included in the RBFDP group.

Author	Type of Study	No of Restorations	Material	Technique	Mean Observation Time	Survival Rate	Success Rate/Failure Rate	Modes of Failure
Kern et al. [16] 2005	PCS	21	In-Ceram alumina, zirconia/Vitadur-Alpha	copy milling/veneered	mean 51 ± 17 months	92.3%		1 fracture
Kern et al. [17] 2011	PCS	22	In-Ceram alumina, zirconia/Vitadur-Alpha	copy milling/veneered	mean 111 ± 44 months	94.4%		1 fracture
Sasse et al. [21] 2012	RCT	30	IPS e.max ZirCAD/IPS e.max Ceram	milled/veneered	mean 41.7 months	100% at 3 y 93.1% at 3 y		2 debondings 1 rotation
Sasse et al. [22] 2013	RCT	30	IPS e.max ZirCAD/IPS e.max Ceram	milled/veneered	mean 64.2 months	100%	failure free rate 93.3%	2 debondings 1 rotation secondary caries
Sailer et al. [23] 2013	RCS	20	IPS e.max Press and IPS Empress/IPS e.max Ceram	pressed/ veneered	60 months	100%	chipping rate 5%	1 chipping
Sailer et al. [24] 2014	RCT	15	IPS e.max ZirCAD/GC Initial	milled/veneered	mean 53.3 ± 23 months	100% at 4 y		2 debondings
Sasse et al. [25] 2014	PCS	39	zirconia/IPS e.max Ceram	milled/veneered	mean 61.6 months	100% 6 y	failure-free rate 94.8% after 6 y success rate 97.4% after 6 y	1 debonding secondary caries
Saker et al. [26] 2014	CCT	20	In-Ceram alumina/	not reported	mean 34 months	90% after 5 y	annual failure rate 0.05%	2 fractures 3 debondings
Klink et al. [27] 2016	PCS	23	zirconia (diff)	milled/veneered	mean 35 ± 15 months	100% at 36 m	success rate 82.4% at 36 m	2 chippings 1 debonding 1 tooth movement
Kern et al. [28] 2017	RCS	108	zirconia	milled/ veneered	mean 92.2 ± 33 months	100%	success rate 92% after 10 y	6 debondings 1 lost restoration
Kern et al. [29] 2017	PCS	22	In-Ceram alumina, zirconia/ Vitradur-Alpha	copy milling/veneered	mean 188.7 ± 47.6 months	95.4% after 10 y 95.4% after 15 y 81.8% after 18 y		2 framework fractures
Naenni et al. [30] 2020	RCT	10	IPS e.max ZirCAD/Initial	milled/veneered	mean 11 y	100% after 10 y		2 debondings
Saker et al. [31] 2020	RCS	20	In-Ceram alumina/VM7	slip casting/ veneered	mean 106.7 ± 24.4 months	85% after 10 y	clinical retention rate 70% after 10 y	3 fractures 6 debondings
Candea et al. [32] 2021	PCS	18 (position not defined)	Zenotec/Zirox Lava Plus/IPS e.max Ceram	milled/ veneered	mean 5.11 ± 1.18 y	100% both anterior and posterior		
Sato et al. [33] 2024	RCS	28	zirconia (unspecified)	unspecified	3 y	91.7%		1 debonding

RCT = randomized clinical trial. PCS = prospective cohort study. RCS = retrospective cohort study. CCT = clinical controlled trial.

**Table 4:** The details of the articles included in the three-unit FPD group.

Author	Type of Study	No of Restorations	Material	Technique	Observation Time	Survival Rate	Failure Rate/Success Rate	Modes of Failure
Probster et al. [34] 1993	PCS	5	In-Ceram alumina Vitradur-Alpha	slip casting/veneered	mean 16.3 months	100%		
Pang et al. [35] 1995	PCS	3	In-Ceram alumina/Vitadur-Alpha	slip casting/ veneered	21 months	100%		localised mild gingivitis (position not clear)
Wolfart et al. [36] 2005	PCS	6	IPS e.max Press	pressed monolithic	mean 48 months	100%		
Taskonak et al. [37] 2006	PCS	12	Empress 2	pressed	24 months		satisfactory rate 50%	2 chippings 4 fractures
Kern et al. [38] 2012	PCS	6	IPS e.max Press	pressed monolithic	60 months	100%		
Sola-Ruiz et al. [39] 2015	PCS	10	Lava/Lava Ceram	milled/veneered	84 months	90%		1 removal (secondary caries)
Alfadhli et al. [40] 2022	RCS	8	KaVo Everest ZS/IPS e.max Ceram	milled/veneered	mean 25 months	87.5%		1 framework fracture
Homa et al. [41] 2025	PCS	16	IPS e.max CAD LT	milled/ veneered	10 y	81.3%	87.5%	1 framework facture 2 removals (pain) 1 chipping

PCS = prospective cohort study. RCS = retrospective cohort study. CCT = clinical controlled trial.

## Data Availability

No new data were created or analyzed in this study.

## Supplementary Materials

The following supporting information can be downloaded at: https://www.mdpi.com/article/10.3390/dj13050219/s1. The PRISMA 2020 checklist is included as supplementary materials, ensuring full transparency in the methodology and study selection process.
